# Chemokines and HIV: The First Close Encounter

**DOI:** 10.3389/fimmu.2015.00294

**Published:** 2015-06-08

**Authors:** Paolo Lusso

**Affiliations:** ^1^Laboratory of Immunoregulation, National Institute of Allergy and Infectious Diseases, Bethesda, MD, USA

**Keywords:** HIV, chemokines, CD8-positive T-lymphocytes, chemokine receptors, antiviral immunity

The first “close encounter” between the fields of chemokines and HIV occurred in the late spring of 1995, when my group at NCI’s Laboratory of Tumor Cell Biology (LTCB) received from Harvard Microchemistry and Proteomics the amino acid sequences of three peptide fragments from an HPLC-purified fraction that we had submitted a few weeks earlier. The sequences showed a perfect match with the human chemokine RANTES/CCL5. The fraction had been isolated from the culture supernatant of an immortalized T-cell clone (FC36.22) producing the elusive “CD8-derived HIV-suppressive factor” whose existence had been postulated by Jay Levy’s group at UCSF since 1986 (1), but whose identity had remained enigmatic for nearly a decade.

Two years earlier, when Fiorenza Cocchi, at that time a post-doctoral fellow from Milan, Italy, had approached me with the proposal to embark on the quest for the “Levy factor,” my first reaction had been anything but enthusiastic. Not that I had any doubt about the significance of identifying an endogenous factor that was believed to help HIV-infected individuals remain asymptomatic by suppressing the virus in a non-cytolytic fashion (2). On several occasions, we had heard Levy illustrate his model, and Bob Gallo, our inspiring lab chief at the LTCB, had often remarked on the importance of resolving this long-standing riddle. However, all attempts to identify the factor until then had failed in the midst of confusion among different designations, experimental models, and mechanistic hypotheses (3), and skepticism about the very existence of such factor was on the rise. Furthermore, my laboratory’s focus was on pathogenesis, with very limited expertise in protein chemistry. And so, we concurred to give it a try, but I made it clear that we would soon drop the project unless we could come up with a robust and reproducible experimental system to justify a long-term commitment.

The original phenomenological observation on the non-cytolytic HIV-suppressive activity of CD8^+^ T cells was made by Chris Walker and Jay Levy in the mid-80s while they were attempting to increase the rate of HIV-1 isolation from asymptomatic seropositive individuals. They found that removal of CD8^+^ T cells from the cultures greatly enhanced their odds of success; when autologous CD8^+^ T cells were added back to the cultures, virus replication was again suppressed but the number of CD4^+^ T cells remained constant, thus ruling out a classic “cytotoxic T-lymphocyte” effect (1). In subsequent years, the same group went on to show that the activity was not bound to MHC restriction and was at least in part mediated by a soluble factor – initially nicknamed “CAF” – that was diffusible through a semi-permeable membrane (4). Moreover, they established a correlation between the levels of CAF production and the asymptomatic state of HIV-1 infection (2), corroborating the clinical significance of this unconventional CD8^+^ T-cell activity. Despite intensive efforts, however, no progress toward the identification of the factor was made over the following years. One of the key challenges was the extremely low level of factor that could be rescued from primary CD8^+^ T-lymphocyte cultures, further complicated by marked donor–donor variability. Yet, Levy insisted that the activity was an exclusive product of primary CD8^+^ T cells and a specific attribute of HIV-seropositive individuals (4), which posed major challenges for production scale-up.

We reasoned that the first critical step to tackle this project was to devise a high-yield and reproducible cellular source for the factor, and we began testing primary and immortalized CD8^+^ T cells from both seropositive and seronegative donors under diverse conditions of activation and culture. The LTCB was an ideal site in this respect because a major focus for over two decades had been the optimization of T-cell growth conditions, culminating in the discovery of “T-cell growth factor,” subsequently named interleukin-2, by Doris Morgan, Frank Ruscetti, and Gallo in 1976 (5), and the isolation of the first human retrovirus, HTLV-1, which can immortalize both CD4^+^ and CD8^+^ T cells *ex vivo*, by Bernie Poiesz and Gallo in 1980 (6). Thus, besides testing primary CD8^+^ T cells, we derived CD8^+^ T-cell lines immortalized with HTLV-1 or its little brother, HTLV-2, and dug deep into the freezers in search for every vintage CD8^+^ T-cell line that we could test. Among the many cells that we screened was 67-I, an HTLV-I-immortalized clone obtained a few years earlier by Anita DeRossi at the LTCB (7), which eventually turned out to be the key to our success. Derived from the peripheral blood of a healthy blood donor, 67-I retained many features of primary CD8^+^ T cells, but unlike the latter it provided a stable and scalable source of soluble factors and was adaptable to grow under serum-free culture conditions, which would eventually simplify purification of the factor.

In parallel to developing an efficient “factor factory,” a second critical need was to establish a highly standardized read-out system for the quantitative determination of antiviral activity. Again, it was essential to overcome the inconsistencies of primary cells and, even worse, of poorly characterized endogenous viral strains harbored by patient-derived CD4^+^ T cells (1, 2). Thus, we embarked in the screening of a wide panel of target cells and viral strains. We eventually opted for PM1, a CD4^+^ clone that we had recently derived from the leukemic T-cell line Hut78, which featured an uncommon susceptibility to diverse HIV-1 variants, including laboratory-adapted and primary isolates with both T-cell and macrophage tropism (8). Taking advantage of this unique quality of PM1, we enhanced our chances of success by entering two divergent HIV-1 variants into our default testing protocol: a typical laboratory strain, IIIB, adapted to grow in continuous T-cell lines, and a macrophage-tropic strain, BaL, passaged exclusively in primary cells, which shared many properties with primary HIV-1 isolates. The system was highly standardized and suitable for high-throughput screening. But when Fiorenza showed up one afternoon with the results of the first experiment, we could hardly believe our eyes: the culture supernatant of 67-I had completely suppressed the BaL strain, while the IIIB strain had continued to replicate impassively. At first, we thought it could only be a technical error, and we decided to independently repeat the experiment in separate laboratories. The results came out a few days later and again they were stunning: in both repeats, BaL was completely inhibited, while IIIB was untouched! Not only did we have in our hands a powerful and reproducible source of HIV-suppressive factor but also the unequivocal bias in favor of the primary-like viral isolate gave us confidence in the specificity of the suppressive effect.

As we had finally pulled together the right experimental tools for the biological side of the project, we set out to identify a skilled protein chemist who could plunge into the backbreaking process of biochemical purification. Thus, we made contact with Tony DeVico, at that time a young research associate at ABL, an NIH-contractor laboratory in Rockville, who had the necessary know-how and enthusiasm to dive into this high-risk/high-reward endeavor. After discussing multiple strategies, we established a basic purification protocol, leaving the option open to modifying it at any time based on the progress of the project.

Looking backwards, although “serendipity” is a term commonly used to describe discoveries in which a “mystery object” remains mysterious until the epilog of the story, never was in my scientific career an experimental design so meticulously and systematically planned ahead in its finest details. This notwithstanding, we were bound for a string of false leads and dead ends that put our trust and determination to serious trial.

Over the next several months, the cycle was repeated over and over: large volumes of serum-free culture fluid conditioned by a high-producer 67-I subclone, FC36.22, were collected, clarified, concentrated by size fractionation, and subjected to HPLC purification using different matrices. Purified fractions were then individually tested against HIV-1 BaL in PM1 cells. Many a cycle was to go “dry,” with no fractions retaining sufficient activity to justify further analysis. Then, a few months down the road, a first intriguing lead: a bioactive fraction containing a single peak was sent for proteomics analysis. Our excitement was sky-high when the results came back a few weeks later showing that the fraction contained human insulin-like growth factor-1 (IGF-1), an immunomodulatory hormone. We immediately attempted to validate the lead, but commercial IGF-1 preparations did not show antiviral activity in our system, and neutralizing antibodies to IGF-1 did not abrogate the activity in crude FC36.22 supernatants. Thus, the lead was abandoned even though IGF-1 was later reported to inhibit HIV-1 (9). After several other “dry” cycles, we stumbled upon another candidate factor, which opened a fascinating new perspective: a bioactive fraction yielded a fragment identical to an HTLV-1 protein, suggesting an intriguing scenario of virus–virus interference. Though captivating as this hypothesis was, the results were not reproducible using concentrated HTLV-1 fractions and, besides, the model was incompatible with the bulk of previous observations made with HTLV-1-negative patient CD8^+^ T cells. We had to move on, and the cycles resumed.

It was a bright and hot late-spring afternoon in Milan. One of those days that give your senses a first savor of the imminent summer: my first one back in Italy after nine and a half years in Bethesda. The phone rang in my temporary office in the new DIBIT building at the San Raffaele Institute where I was creating my own Laboratory of Human Virology. When I picked up, Fiorenza’s voice on the other side could hardly conceal her excitement: “We’ve got new sequences – she said right off the bat – It’s RANTES!”. While the call was still on, I jumped on MEDLINE and crossed the two keywords: “RANTES” and “HIV.” I hit “return” and, to my astonishment, the result was … zero! Even just by chance, almost any two keywords yield at least a half dozen citations. “Zero” was not only really amazing but also somewhat frightening. It was like in those science-fiction movies when they open a door in a dark empty hallway to find themselves into the dazzling light of a totally new dimension: there we were, all of a sudden projected into the fantastic world of the chemoattractants!

Of course, having chased several false leads in the previous months made us temper our enthusiasm and keep our feet on the ground. But a few weeks later, when we received the sequences from a second bioactive fraction matching 100% the “sister” chemokine MIP-1α/CCL3, our adrenalin level had a dramatic jolt. We knew that this time we were on the right track. We swiftly ordered a set of recombinant proteins and neutralizing antibodies, including those specific for the third “sister” chemokine, MIP-1β/CCL4, even though we had not yet received proteomics confirmation for this last member of the trio. In early September, we submitted an article to *Science* that, in spite of a single hopeless negativist referee who raised all sorts of questions about the past, present, and future relevance of our findings, was rapidly accepted and appeared in print on December 15, 1995 (10). With an unorthodox move, *Science* heralded our paper with a Commentary by Michael Balter in the December 8 issue (11), concomitant with the publication in *Nature* of another candidate CD8-derived antiviral factor, interleukin-16 (12). In the paper, we presented conclusive evidence that: (i) RANTES, MIP-1α, and MIP-1β are three potent and specific endogenous HIV-1 inhibitors; (ii) they are abundantly produced by both immortalized and primary CD8^+^ T cells; and (iii) altogether they constitute a major component of the soluble HIV-suppressive activity produced by these cells (10). For the first time, we were exposing the “double life” of certain chemokines, turned overnight from aseptic cellular-traffic policemen into specific endogenous virus-busters. One of the greatest surprises in this saga was the realization that the long-sought-after CD8 anti-HIV factor in fact, comprises multiple factors, breaking a central dogma of the original model (4). Indeed, evidence continues to accumulate on the existence of a wide range of endogenous HIV-suppressive factors, as illustrated by our recent identification of XCL1/lymphotactin as a novel anti-HIV chemokine produced by CD8^+^ T cells (13).

The rest of the story is well known. Almost fictional was the extraordinary time coincidence whereby <6 months after the publication of our paper Ed Berger and his colleagues at the NIAID reported in *Science* the first HIV-1 coreceptor, fusin/CXCR4 (14) – another “serendipitous” discovery? – which happened to be an orphan “chemokine” receptor originally identified by Bernhard Moser’s group in Bern (15). Likewise, virtually at the same time, Phil Murphy’s group at the NIAID (16) and Marc Parmentier’s group in Brussels (17) were both characterizing the same novel “chemokine” receptor (CCR5) specific for RANTES, MIP-1α, and MIP-1β, which was almost immediately shown by Berger’s and four other groups to be the second, physiologically most relevant, HIV-1 coreceptor (3). Curiously, despite having worked on the same campus for years, I had never previously met Ed or Phil, who later became good friends of mine and, in the case of Ed, a close collaborator. The extraordinary convergence and synergy among these independent discoveries, collectively saluted by *Science* as one of the “breakthroughs-of-the-year” at the end of 1996 (18), inaugurated a new era of AIDS research, triggering a chain-reaction of additional breakthroughs which altogether dramatically accelerated our understanding of HIV physiology and pathogenesis, and posed the foundations for new therapeutic and preventive strategies with far-reaching consequences for the ultimate control of the HIV/AIDS pandemic.

## Conflict of Interest Statement

The author declares that the research was conducted in the absence of any commercial or financial relationships that could be construed as a potential conflict of interest.
